# Economic recession and health of the people

**DOI:** 10.4103/0972-2327.48844

**Published:** 2009

**Authors:** Sanjeev V. Thomas

**Affiliations:** Professor of Neurology and Editor, Annals of Indian Academy of Neurology, Department of Neurology, SCTIMST, Trivandrum, India. E-mail: editor@annalsofian.org

Japan had passed through an economic recession in the 1990s, when their Gross Domestic Product (GDP) growth dropped from + 5.5 to −.5, during which period the male suicide rate reached a peak 18.8–25.2. This increase was due to the ‘epidemic’ of suicides among working class males and was attributed to the rapid changes in industrial structure and working environment that followed recession. Further, the prevalence of coronary risk factors had risen in this subpopulation during the recession. A more detailed analysis and survey had shown that the risk of self-perceived poor health was significantly more for the lower income menial job group than for the higher income managerial group. This confirms what is apparently obvious – the poor and marginalized are more vulnerable to the economic decline. This is rather unexpected from a country known for its sound social security system, compulsory pension, and high standards of health for several decades.^1^ An analysis of the economic profile and mortality patterns in Sweden over three decades had shown that economic growth plays a principal role in reducing mortality at nearly all ages, and specifically mortality due to total cardiovascular disease, cerebrovascular disease, total heart disease, ischemic heart disease, total malignancies, disorders of infancy, and motor vehicle accidents. Economic recession, by contrast, is related to increases in total mortality for virtually all age groups, in both sexes, for major causes of death and causes due to psychopathological conditions.^2^ India and the rest of the world are now in a whirlpool of economic recession. How is it influencing our health and our march to attain the millennium goals by 2015? The economy in India had been growing remarkably in the recent past. We had made significant progress in healthcare, health delivery system. These strides in health scenario were not based on the private and public initiates in healthcare. Nevertheless, what is worrying is the relative lack of growth in human resources to support optimal health delivery system in India. The investment in healthcare and delivery system is likely to grind to a halt from the already depleted state. Extrapolating from the experiences of similar situations in the past there would be marked increase in the incidence of stroke, demyelinating diseases, malignancies, and several other neurological disorders besides the increase in infections. How do we prepare ourselves to handle this additional burden and threat? It is possible to reduce its impact by judicious redeployment of our resources? The academy as an institution and doctors as individuals need to be vigilant about these dynamics. There need to be better dialogue between administrators, economists, scientists, and clinicians to develop strategies to handle these emergencies. I would like to invite articles and view points on this issue for publication in this journal.

White matter diseases are increasingly recognized in India. Increased awareness of these conditions, availability of sensitive MRI services that could identify them, and opportunities for newer treatments have probably influenced this increased detection rate. It is unclear, whether there is an increase in the incidence of white matter diseases in India. Socioeconomic transition, environmental changes, and population migration could also be contributing to these changes. In this issue, we have presented a review article on a practical approach to diagnose various white matter diseases.

Proteomics is a new branch of science that studies the proteins in toto for its structure and functions. Proteomics opens a window into the synthesis, transformations, and kinetics of proteins which are important in cell development and cell-to-cell signaling. Proteomics can be applied to study certain proteins as biomarkers in conditions like Alzheimer's disease. The commonly used techniques for such studies include western blot, immunohistochemical staining, enzyme linked immunosorbent assay (ELISA), or mass spectrometry. We have presented one review on recent developments in proteomics with reference to mass spectrometry. This technique is slowly becoming available in India. There is immense scope to set up long-term studies on proteomics for diagnostic purposes in India.

We are glad to present one detailed technical note on EEG examination of neonates. Neonatal EEG is a challenge even for experienced electroencephalographers. Small babies, particularly preterm and small for date, are an enormous challenge to the technologists to make good recording. Deviations from normal development, structural and anatomical disturbances, and metabolic changes make interpretation of these traces very difficult and often misleading. A systematic approach to the recording techniques and steps in interpretation are provided in this technical note. I hope that most readers who are involved with the care of newborns would find this very helpful.

Our journal has made substantial growth in the past three years. The standards of the journal in content turnout and visibility have improved remarkably during this period. It is now indexed with several prestigious databases. This credit goes to all the members of the editorial board, editorial advisory committee, and the academy who had supported it so well. The Medknow publishers had done a remarkable job to bring out this journal both in print and online in a timely manner. We received unrestricted help from all the experts who had taken time from their busy schedule to review the articles for us. The contributors deserve special thanks for choosing this journal to publish their articles and for complying with our requirements. We had received unrestricted support from several healthcare industries that had accepted our open policy and gave their advertisements. Several institutions have started new subscription to our journal. I would like to thank each and every one of them for their strong support and assistance but for which we would not have achieved this growth. I accept with all humility the extension of my term as Editor of this journal for another three years which is indeed a matter of great pride and honor. The new editorial board and editorial advisory committee have come in to place from this issue of the journal. I am confident that we could do a good job with all their support.
